# Transparent Antibacterial Nanofiber Air Filters with Highly Efficient Moisture Resistance for Sustainable Particulate Matter Capture

**DOI:** 10.1016/j.isci.2019.07.020

**Published:** 2019-07-19

**Authors:** Hui Liu, Jianying Huang, Jiajun Mao, Zhong Chen, Guoqiang Chen, Yuekun Lai

**Affiliations:** 1 National Engineering Research Center of Chemical Fertilizer Catalyst (NERC-CFC), College of Chemical Engineering, Fuzhou 350116, P. R. China; 2 National Engineering Laboratory for Modern Silk, College of Textile and Clothing Engineering, Soochow University, Suzhou 215123, P. R. China; 3 Department of Chemical and Biomolecular Engineering, University of California, Los Angeles, CA 90095, USA; 4 School of Materials Science and Engineering, Nanyang Technological University, 50 Nanyang Avenue, 639798 Singapore, Singapore

**Keywords:** Materials Characterization, Nanomaterials, Pollution

## Abstract

Particulate matter (PM) pollution has posed great threat to human health. This calls for versatile protection or treatment devices that are both efficient and easy to use. Herein, we have rationally designed a novel reusable bilayer fibrous filter consisting of electrospun superhydrophobic poly(methylmethacrylate)/polydimethylsiloxane fibers as the barrier for moisture ingression and superhydrophilic chitosan fibers for a PM capture efficiency of over 96% at optical transmittance of 86%. Furthermore, it could realize a high-level PM_2.5_ capture efficiency (>98.23%) even after 100-h test during extremely hazardous air environment (PM_2.5_ > 3,000 μg m^−3^) and retain a high PM removal efficiency (PM_2.5_ > 98.39%) after five washing cycles. Besides, such membranes possessed high antibacterial activity at 96.5% for *E. coli* and 95.2% for *Staphylococcus aureus*. As a proof-of-concept study, continuous particle removing has been successfully demonstrated on a window screen to prevent particle pollution.

## Highlights

• The superhydrophilic chitosan fibers play the role of enhanced removal capability
• Superhydrophobic PDMS/PMMA fibers prevent water accumulation inside the membrane
• Nanofibrous filters present excellent antibacterial property due to the chitosan component
• Robust filters exhibit good recyclable performance for sustainable PM capturing

## Graphical Abstract

**Figure:**
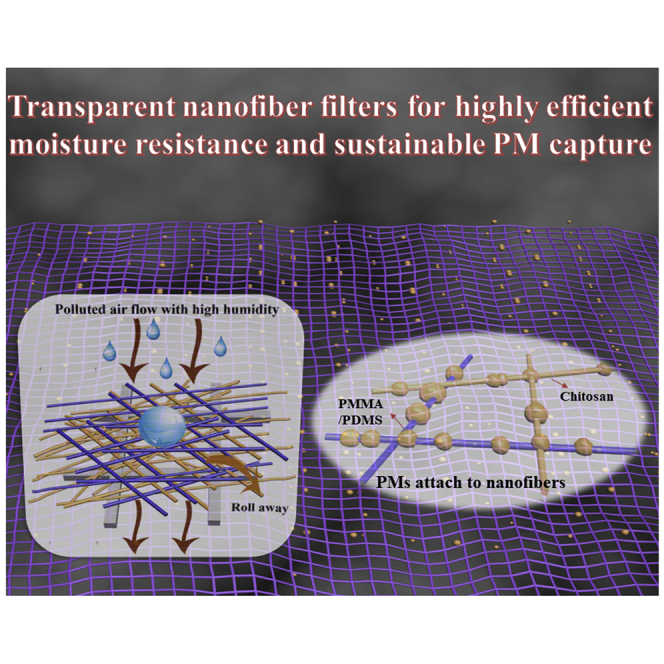

## Introduction

Air pollution by particulate matter (PM) has posed a tremendous threat to human health and living quality (Gao et al., 2017, Wu et al., 2017, Zhang et al., 2013). PM, complexly composed of extremely tiny liquid droplets and solid particles, is capable of penetrating profoundly into the lungs and then reaching the alveolar region, leading to lethal condition and increasing morbidity and mortality in case of long-term exposure (He et al., 2016, Horton et al., 2014, Li et al., 2016, Mahowald, 2011, Meng et al., 2016, Uyar et al., 2010, Xie et al., 2015). In general, PM particles can be categorized based on aerodynamic equivalent size, ranging from several nanometers to tens of micrometers. Accordingly, PM_2.5_ and PM_10_ are defined as particle diameter less than 2.5 and 10 μm, respectively. To alleviate the problems related to PM pollution, capturing of PM particles is a straightforward solution, particularly the more dangerous PM_2.5_ particles, which are composed of inorganic (such as SiO_2_, SO_4_, and NO_3_) and organic matter (organic carbon and elemental carbon). They are usually derived from various sources such as industrial emission, biomass burning, secondary aerosols, coal combustion, vehicular exhaust emission, and soil dust (Huang et al., 2014, Lelieveld et al., 2015, Mackey et al., 2013, Platt et al., 2014, Zhang et al., 2018b). The differences in the behavior of PMs are mostly determined by their surface morphology and chemical composition. For rigid inorganic PMs, the main capturing method is to intercept using a filter. On the other hand, soft particles containing carbon compounds and water coming from exhaust emission would strongly bind to the filter surface and may change their shape through deformation during the capture process. Therefore the surface properties of the filtration tools are of great importance for enhancing the removal of PM particles and thus should be carefully designed.

In the past, attention has been mainly focused on individual protection in the outdoor space, like face masks, etc. Less attention has been paid to protection in indoor buildings, except the central air-conditioning or ventilation systems in some modern commercial buildings. Residential housings lack sufficient protective filtration tools to keep the air clean and healthy (Khalid et al., 2017, Liu et al., 2015, Zhang et al., 2016a). A variety of techniques and materials have been explored for PM filtration (Chen et al., 2017, Jeong et al., 2017, Jung et al., 2018, Mao et al., 2019, Zhang et al., 2016d, Zhang et al., 2017b, Zhu et al., 2017). Two types of filtration have been extensively utilized so far: the porous filters and the fibrous filters. The latter has the advantages of being easy for mass production, cost effective, and energy efficient. However, the traditional fibrous filters, including the spun bonded fibers, glass fibers, and melt-blown fibers, suffer from various drawbacks caused by the relatively large diameter of several micrometers: bulky, low quality factor (QF), and weak capture ability for fine particles. To meet the requirements for transparent filters with high efficiency for PM particle removal, electrospinning technique for PM removal was demonstrated by using various fiber filters including polyacrylonitrile (PAN), polyvinylpyrrolidone, polystyrene, polyvinyl alcohol, and polypropylene as a filtration comparison (Liu et al., 2015). PAN was revealed to possess the best capture ability. Subsequently, polyimide nanofibers, which have high thermal stability, were found to possess a high PM_2.5_ removal efficiency at temperatures up to 370°C (Zhang et al., 2016a). Besides, Ko et al. have demonstrated a Ag nanowire percolated network as a transparent PM filter with a very high particle removal efficiency. The fabricated filter had excellent durability and reliability and thus could be reused for multiple times after a simple cleaning process. Moreover, Ag nanowire has an intrinsic antibacterial activity to kill any harmful microbes. This research has paved way for multifunctional filtration materials (Jeong et al., 2017).

Water vapor generated in a high-humidity environment tends to cause obstruction of the filter pores. Unfortunately, most current existing filter materials suffer from high air resistance induced by the direct contact of air molecules with the membranes under high humidity, subsequently resulting in low removal efficiency. Only very few works were presented to solve this problem. Ding et al. (Zhao et al., 2017a) designed a gradient structure from hydrophobic to superhydrophilic by introducing PAN/silicon dioxide fibers to act as the moisture vapor delivery vector and hydrophobic polyvinylidene fluoride fibers to serve as the repellent component to avoid forming capillary water in high humidity. Besides, a bilayer triboelectric air filter with hydrophobicity has been rationally designed by Wang et al., which could maintain consistent PM_2.5_ removal efficiency in high-humidity condition (Bai et al., 2018). In general, to maintain the filter working efficiently in a high-humidity environment, certain degree of hydrophobicity is required. The water molecules from air were able to accumulate on the surface of the superhydrophobic fiber filters to form bigger drops, and subsequently roll away without forming a water film. As we all know, PMMA nanofibers are hydrophobic because of the terminal ester group. To realize superhydrophobicity, materials with low surface energy have to be used. Cui et al. (Khalid et al., 2017) have investigated the filtration performance of pure PMMA nanofibers, which presented unsatisfactory removal efficiency. Therefore, chitosan, a kind of polysaccharide from chitin with biodegradability and without toxicity, has been chosen to enhance the removal ability because of its functional groups. Besides, chitosan is excellent in some properties such as its inhibition of the growth of bacteria, yeasts, and fungi. More importantly, it is very easy for chitosan to be polarized due to its strong polarity, which will significantly benefit the effective adsorption of PM particles on the chitosan nanofibers. All these favorable property attributes make chitosan a promising candidate for PM filtration. Long et al. (Zhang et al., 2017a) have demonstrated the *in situ* electrospinning of chitosan solution in an enclosed environment using an exquisite self-designed spinning device. High removal efficiency was realized due to the cooperation of the surface adhesion and the strong electrostatic adsorption. However, such *in situ* electrospinning process may not be suitable for a real environment in practical use. So, for the first time, we used the obtained robust chitosan fibrous membranes for sustainable high-efficiency PM removal.

In this work, we present a novel strategy for developing a transparent multilayer nanofibrous filtration membrane for efficient PM_2.5_. The superhydrophobic poly(methylmethacrylate)/polydimethylsiloxane (PMMA/PDMS) fibers contribute to water moisture transfer, whereas superhydrophilic chitosan fibers are responsible for high PM removal. Polar functional groups and nanometer-scale diameter have jointly paved the way toward outstanding filtration efficiency. Such filters with 54% optical transmittance could maintain a stable high removal efficiency, air drop pressure, and flow rate. Furthermore, after 100-h test during hazy air condition (PM_2.5_ > 3,000 μg m^−3^), a high-level capture efficiency could still remain at 98.23% for PM_2.5_. Besides, this membrane possesses excellent antibacterial property. Taking practical use into consideration, post-adsorption separation of PM particles has been demonstrated. This work is expected to open a new avenue for the next-generation fibrous filters for efficient and sustainable treatment of air pollution under harsh working conditions.

## Results and Discussion

### Design, Structure, and Mechanism of Multilayer Nanofibrous Membranes

**Figure 1:**
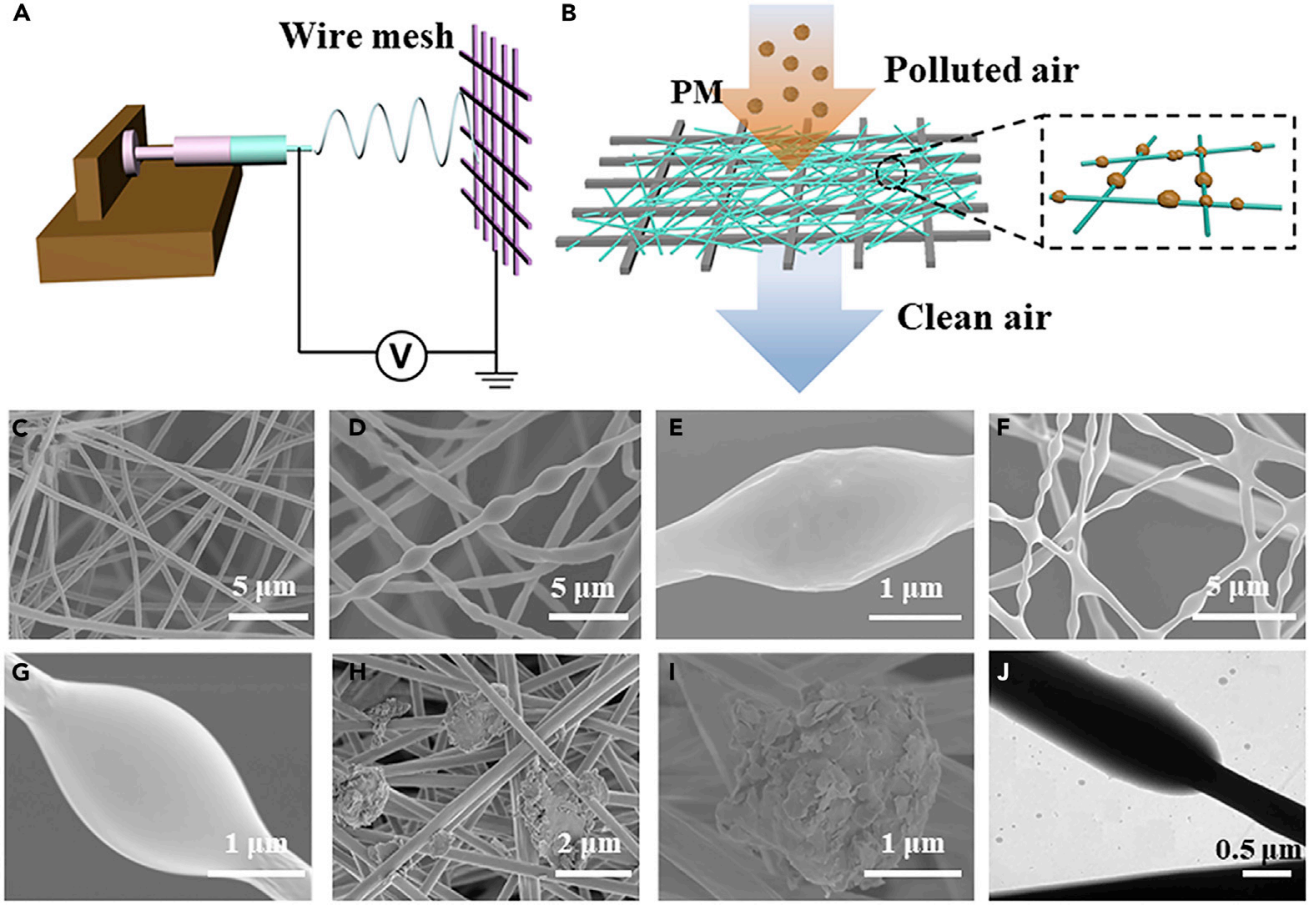
Fabrication Process and Morphological Characterization of Air Filters (A) Schematics of fabricating air filters by electrospinning. (B) Schematic illustration of the filtration process of fibrous membranes. (C) SEM images of air filters before filtration. (D and E) PMMA-PDMS fibers after filtration from burning incenses, at different magnifications. (F and G) Chitosan fibers after filtration from burning incenses, at different magnifications. (H and I) SEM images of air filters with captured dust particles, at different magnification. (J) TEM images showing the morphologies of PM particles captured on fibers.

Electrospinning (Khalid et al., 2017, Liu et al., 2015, Zhang et al., 2016a), as a versatile processing method, has been applied to produce uniform nanofiber filters from diverse polymer solutions with controllable dimensions (Figure 1A). To synthesize uniform nanofibers, selection of an appropriate solution concentration, voltage, and distance between the tip of a syringe and the supporting collector is of great importance. Wire meshes were applied here as collecting substrate. On account of the distribution of given electrical field, the polymer solution was pulled into nanofibers and then lay across the mesh holes, thereby forming network as an air filter. Here, smoke from the burning incense, as an ideal model system, has been selected as the main PM source. Such incense smoke contains extensive PM particles possessing a variety of sizes, and it comprises various pollutant gases existing in the haze, including SO_x_, NO_x_, and CO_x_; some typical volatile organic chemicals, for instance, polycyclic aromatic hydrocarbons, aldehydes, xylenes, toluene, and benzene; as well as some other contaminants (Lin et al., 2008). Besides, the concentration of particles with a diameter of 0.3 μm was three orders higher than that of particles with a diameter of 10 μm in incense smoke. The concentration distributions of the PM particles indicate that approximately 90% of them are smaller than 1.0 μm and can deposit directly into human alveoli (Zhang et al., 2018b).

In this work, we aim to produce nanofibrous filters possessing moisture transfer ability and effective adsorption of PM particles. Such functional filters were designed on the basis of criteria that the filtration materials should comprise superhydrophobic components to avoid the sharp growth of air resistance during high humidity, as well as contain functional fibers to ensure high PM capture ability. PDMS/PMMA composite fibers were selected as the superhydrophobic component because of the low surface energy from identical terminal groups (methyl) (Liu et al., 2017). Also, the main adsorbing role was played by chitosan fibers ascribed to their strong polarity exhibited by the nitrile chemical groups (Bhattarai et al., 2005, Zhang et al., 2017a). Besides, these two fibers possessed ultrathin diameters of about 300 nm, which is potentially beneficial for enhancing the PM capture ability. And PM pollution filtration was carried out in a home-built device, as shown in Figure S3.

Figure 1B presents the filtration mechanism. Once hazy air enters into the fibrous percolation structure, the pollution particles would be trapped in the random network and have strong affinity with the nanofibers, leaving the purified gas flowing out. We have applied scanning electron microscopy (SEM) and transmission electron microscopy (TEM) to investigate the fiber filters before and after adsorption and characterized the morphology to reveal fundamental interactions between the fibers and the PM particles. In Figures 1C–1G, the SEM pictures show that these two types of nanofibers have almost identical morphology after the PM particle attachment. A careful examination finds that these PM particles tightly wrapped up the fiber surfaces in an irregular ellipsoidal shape, whereas the nanofibers before filtration looked smooth. The TEM image in Figure 1J also suggests that the PM particles captured by the fibers have an amorphous carbon-like structure with the external layer containing light organic matter. Besides, investigation on the surface chemistry has been conducted via Fourier transform infrared (FTIR) spectroscopy (Figure S2), which displayed enhanced peaks of C=O, C-O functional groups, and newly emerging C-N groups at ∼1,757, 1,238, and 1,386 cm^−1^ respectively, after the PM capture. C, N, and O elements were detected in the energy-dispersive X-ray spectroscopic (EDS) images (Figure S1). The consistent results from above two analyses suggest that the smoke was composed of mostly organic carbon with different polar functional groups like aldehyde, alkanes, and so on. The highly polar functional groups such as C–N, C=O, and C–O are mainly distributed on the outer surface of the PMs. The chitosan fibers with nitrile chemical polar groups and a high dipole moment have stronger dipole-dipole and induced-dipole intermolecular forces with these functional groups, so that the PM particles could be efficiently captured by Columbic forces.

The PM removal efficiencies of PDMS/PMMA-chitosan nanofibrous filters with diverse composite proportions have been presented in Figure S4A. With increasing content of chitosan fibers in the composite nanofibers, there was an increasing trend in the removal efficiency. The PM_2.5_ removal efficiencies of PDMS/PMMA-chitosan filters with ratio between PDMS/PMMA and chitosan fibers of 4/0, 3/1, 2/2, 1/3, and 0/4 were 85.27%, 89.96%, 94.54%, 99.18%, and 99.66%, respectively. The enhanced polar surface chemistry from chitosan fibers and ultrathin dimension has contributed to the enhancement in removal efficiency. On the basis of the similar removal efficiency and existence necessity for PDMS/PMMA component for other functional considerations (discussed later), the proportion of 1/3 was set as an optimized filter composition.

We have also tested soil dust particles to investigate the adsorption of solid aerosol particles on the fiber filters, as shown in Figures 1H and 1I. The soil dust was milled into nanosized particles before being tested in the same manner as the incense particles. It was found that once the dust particles were in contact with the fibers, they were immediately attached to and accumulated on the fiber surface, forming dendrite-like morphology (Zhang et al., 2018a). This test has confirmed the outstanding filtration capability of diverse types of PMs.

### PM Capturing

**Figure 2:**
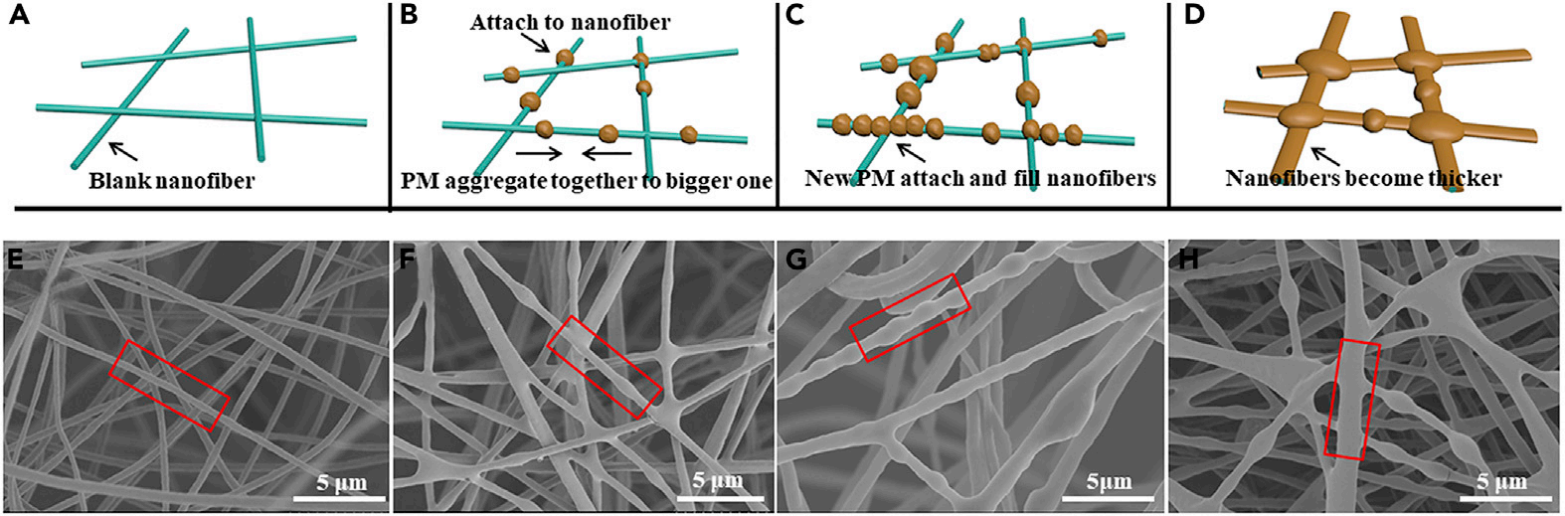
PM Capturing Process (A–H) (A–D) Schematics corresponding and (E–H) SEM images displaying the capturing process of PM particles by nanofiber filters at different stages. (A and E) the blank nanofiber; (B and F) more and more PM particles attached to the nanofibers and agglomerated to larger size; (C and G) the new PM particles directly attached to the old particles and filled the fiber space; (D and H) the nanofibers turned thicker and the diameter increased significantly.

Investigation of the capturing process at different time sequences has been illustrated in Figures 2A–2H (Liu et al., 2015). Figures 2B and 2F have displayed the initial capturing stage, at which the PM particles first bound strongly on the nanofibrous filters. With continuous feed of the incense smoke, the nanofibers were attached with more and more PM particles. Furthermore, the PM could move along the nanofibers and merge together as bigger ones, thereby leaving behind much empty room for adsorbing new particles (Figures 2C and 2G). In addition, the incoming PM particles were also able to directly attach to the old particles and agglomerate to larger size. With the capturing process in progress, the agglomerated larger particles have filled the fiber space. Particularly, more and more particles could accumulate at the junction of filters and then become stabilized spherical shapes of larger sizes. As this adsorption duration increases, the nanofibers obviously turn thicker and the diameter has increased significantly (Figures 2D and 2H).

### *In Situ* Investigation of Transition Behavior of Air Flow under High Humidity

**Figure 3:**
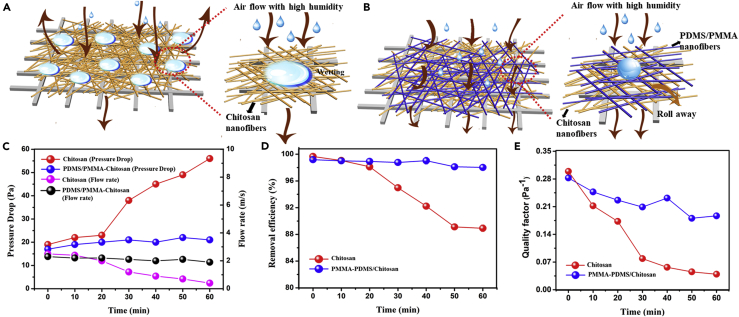
*In Situ* Investigation of Transition Behavior of Air Flow under High Humidity (A and B) Schematic illustration of air flow pass through (A) pure chitosan nanofiber filters and (B) PDMS/PMMA-chitosan nanofiber filters under humidity conditions. (C–E) (C) Pressure drop and flow rate, (D) removal efficiency, and (E) quality factor of pure chitosan nanofibers and PDMS/PMMA-chitosan nanofiber membrane at different times under humidity condition of 300 mL h^−1^.

Schematic illustration of the interaction between the filters and vapor-containing air flow is displayed in Figures 3A and 3B. Water vapor in the air under high-humidity condition was easy to condense on the chitosan nanofiber surfaces due to the capillarity and the hydrophilicity of these nanofibers, which would remarkably affect the continuous airflow. Gradually, the accumulated micro-water droplets on the pristine chitosan fibers would lead to the formation of water films on the filters. For further verification, we have put these two membranes in high-humidity environment for 1 h, the pure chitosan was completely wet and full of water, whereas the PDMS/PMMA-chitosan composite membrane showed almost no change on the surface (Figures S6A and S6B). As shown in Figures 3C and 3A sharp increase of the pressure drop from 19 to 56 Pa and obvious fall of flow rate from 2.5 to 0.4 m s^−1^ have occurred after 1 h under high humidity condition of 300 mL h^−1^. The removal efficiency has decreased from 99.66% to 88.92% (Figure 3D). When the composite PDMS/PMMA-chitosan fiber membrane was used, the water molecules from air were able to accumulate on the surface of the fiber filters to form bigger drops, and subsequently roll away, owing to the superhydrophobicity (contact angle at 165.6°) of the PDMS/PMMA nanofibers with a low adhesive force of 12 μN (Figure S4B) (Tang et al., 2013). The airflow was capable of passing across the filter, resulting in a minor pressure drop and flow rate. As shown in Figure 3E, the pressure drop for PDMS/PMMA-chitosan fibers was around 17 to 21 Pa and flow rate dropped slightly to 1.9 m s^−1^. Remarkably, the removal efficiency was very stable (Figure 3D), reflecting the key role of superhydrophobic PDMS/PMMA fiber layer in a high-humidity condition. The QF has also been calculated to determine the overall filtration performance of the filters. It was defined as the following equation: QF = −ln(1−η)/Δp, where Δp and η are the pressure drop and removal efficiency, respectively. As shown in Figure 3E, the QF values of pure chitosan displayed a sharp decrease, whereas those of PDMS/PMMA-chitosan fibers declined slowly and still kept with a higher value.

### Transparent Nanofiber Filters

**Figure 4:**
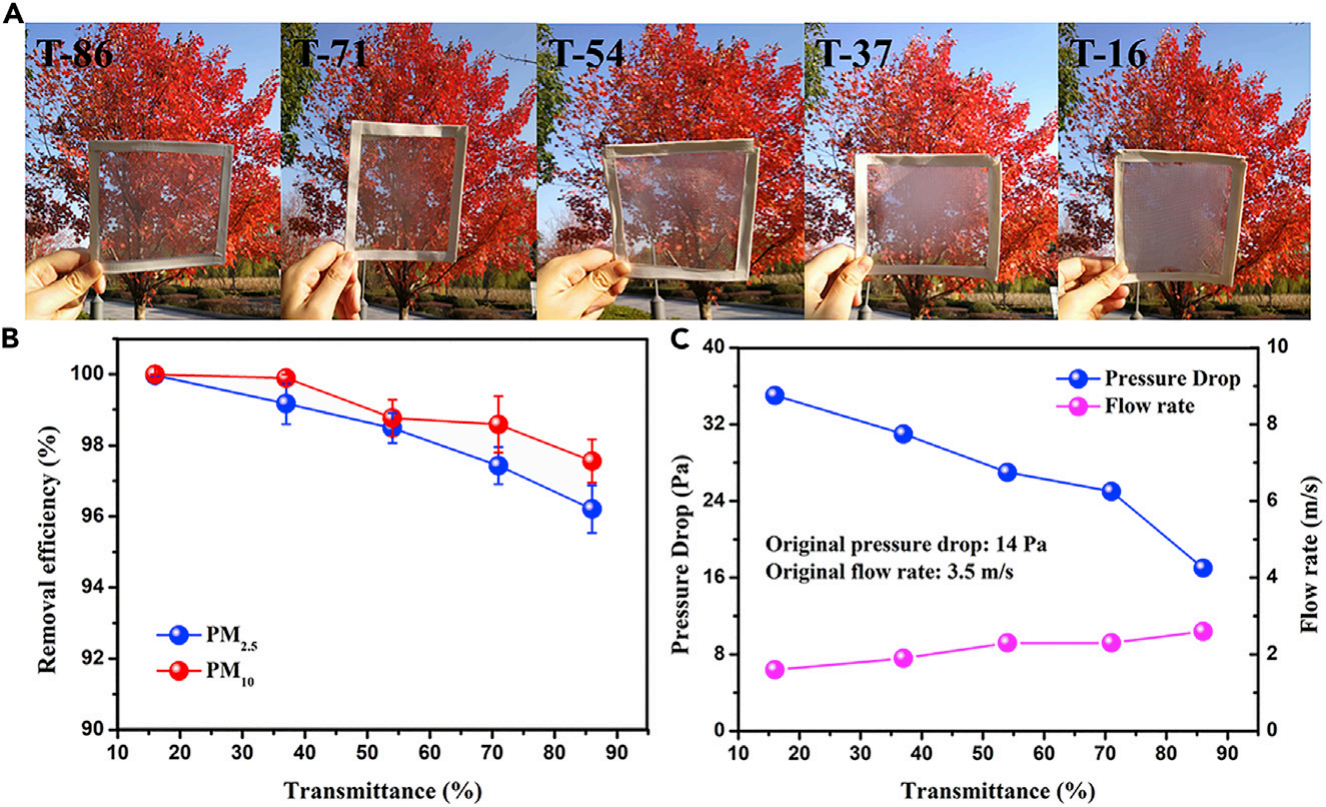
Transparent Nanofiber Filters (A) Photographs of PDMS/PMMA-chitosan transparent air filters at different transparencies. (B and C) (B) PM_2.5_ and PM_10_ removal efficiency and (C) pressure drop and flow rate of transparent filters at different transmittances.

Other than the adsorption performance, the filter's ability for visible light transmission (Huang et al., 2019) is another key factor that should also be taken into consideration, for example, when it is applied for household windows. Photographs of nanofiber filters with different optical transmittance, at 16%, 37%, 54%, 71%, and 86%, are presented in Figure 4A. An in-depth study indicates that opulent sunshine could penetrate through the nanofiber filters and that it was very clear to view the colorful image when transmittance was more than 50%. However, with further increase of the thickness for fibrous membranes, the light transmission was much reduced, thus resulting in a blurry view. Therefore, it is possible to coat window mesh at an optimum transmittance level so that there will be reasonable light transmission, whereas PM particles are effectively blocked. Figure 4B reveals that the removal efficiency for PM_2.5_ and PM_10_ decreases when the transmittance level of filters increases. Nevertheless, an outstanding capturing efficiency of more than 96% at optical transmittance ranging from 16% to 86% has been successfully achieved. At transmittance of 54%, the removal efficiency was up to 98.49%. In addition, air flow is another significant parameter for air filters. The pressure drop and flow rate of the fiber filters with various levels of transmittance have been quantitatively analyzed, and the results are shown in Figure 4C. A trend of increasing pressure drop and decreasing air flow rate with the decreasing transmittance is obvious. Compared with a blank wire mesh with an original pressure drop of 14 Pa and flow rate of 3.5 m s^−1^, fibrous filters with 86% transmittance showed a smaller change. Besides, we have compared the filters in this work with other reported fibrous filters (Li et al., 2018a, Li et al., 2018b, Liu et al., 2015, Wang et al., 2014, Zhang et al., 2016a, Zhang et al., 2016b, Zhang et al., 2016c, Zhao et al., 2017a, Zhao et al., 2017b, Zuo et al., 2017), which clearly shows that PDMS/PMMA-CS (chitosan) nanofiber filters have the best air filtration performance considering PM removal efficiency, pressure drop, and the QF (Figure S5).

### PM Adsorption and Desorption

**Figure 5:**
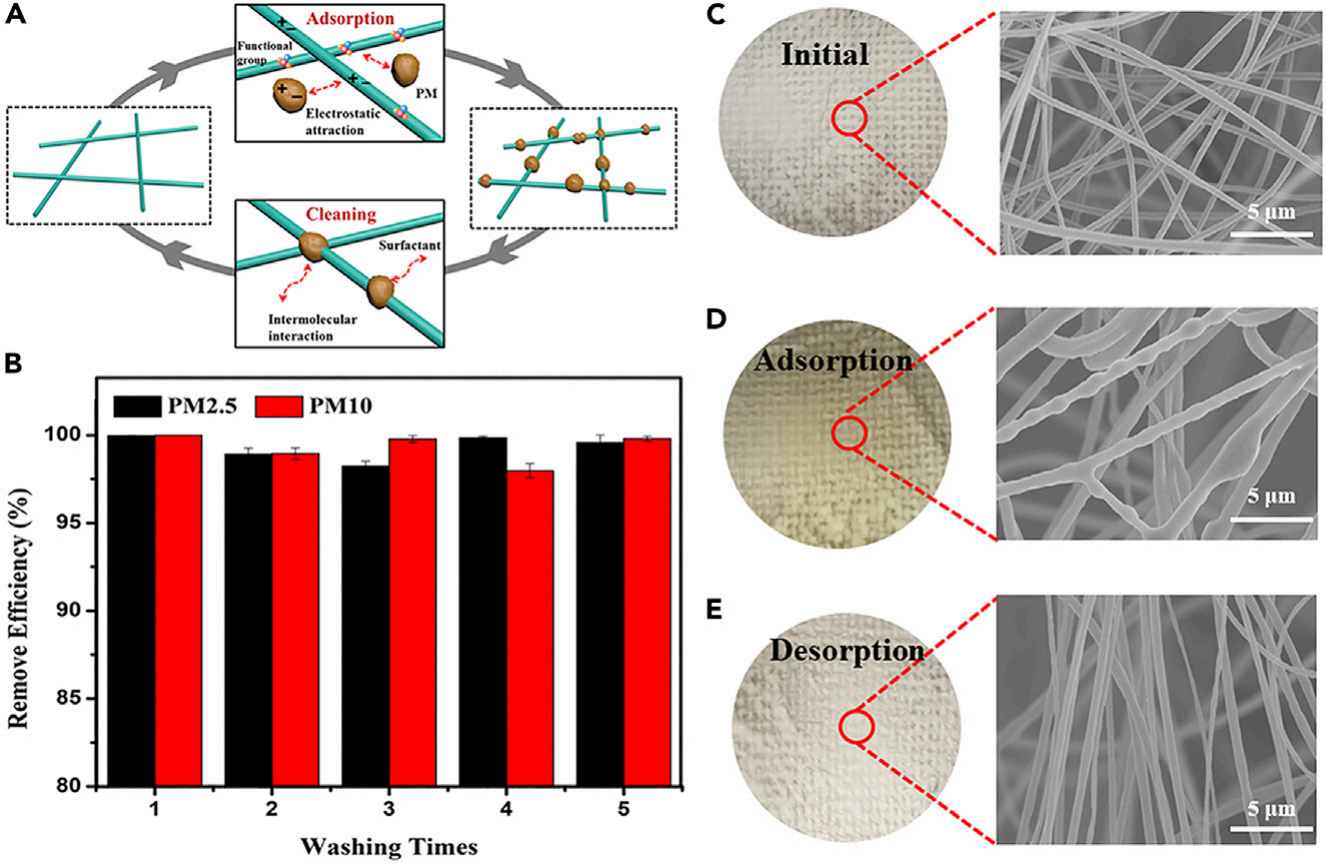
PM Adsorption and Desorption (A) A schematic explanation for the mechanism of PM adsorption and desorption by nanofiber filters. (B) The removal efficiency of as-fabricated nanofiber filters after washing five times. (C–E) (C) Photographs and SEM images of the initial filters, (D) filters after adsorption, and (E) filters after desorption.

The PDMS/PMMA-chitosan filters were easy to clean by water with general detergent at 45°C for 10 min. Here, an air filter at 16% transmittance was applied for this test. The mechanism for PM adsorption and desorption is schematically shown in Figure 5A (Jeong et al., 2017). First, the PM particles generated from the incense smoke were collected by the nanofibers because of the high specific surface area and strong electrostatic attraction due to the presence of functional groups. During the cleaning of the fiber filters, intermolecular interaction between PM particles and dipole detergent was high enough to overcome electrostatic force, thus separating the particles from the fibers. The photographs and SEM images in Figures 5C–5E have revealed the surface morphologies of the fibrous filters at the initial, adsorbed, and desorbed states. The fiber surface of initial membranes appears white and is very smooth. After taking up the incense smoke, the filters turned yellow. The color returned to its initial white appearance after the PMs have been removed upon cleaning. Also, the structure of the fiber membranes remained stable; there was no fiber breakage or collapse observed after the washing process. In addition, the similar content of N element as the initial fibers based on the EDS results and the weakening peaks of C=O, C-N, and C-O in the FTIR after the washing process both indicate the complete removal of PMs from the nanofibers. Figure 5B has also displayed the removal efficiency of PDMS/PMMA-chitosan filters after cleaning several times. High adsorption efficiency over 99% was maintained after five washing cycles, proving its excellent washability.

### Mechanical Durability and Long-Term Performance

**Figure 6:**
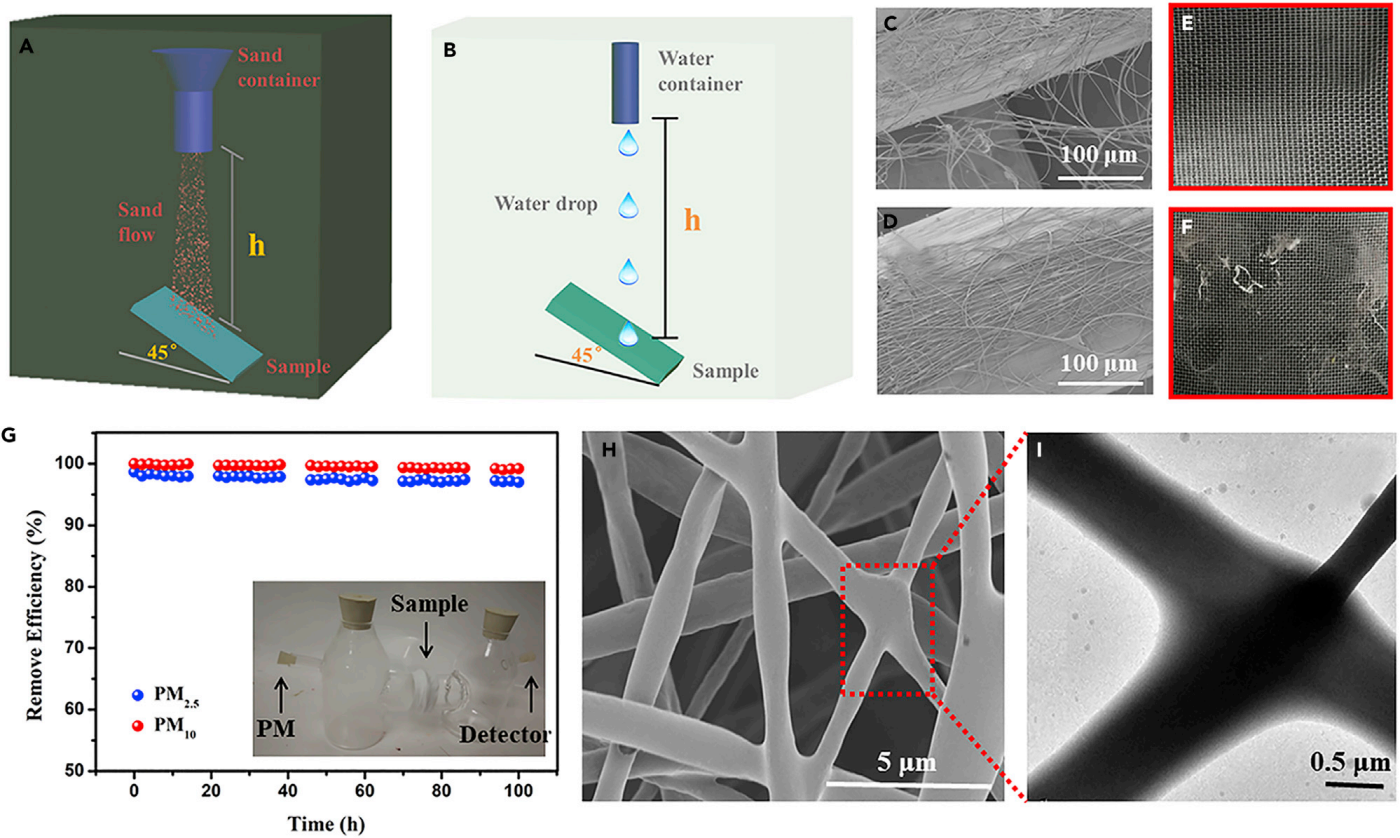
Mechanical Durability and Long-Term Performance (A–F) (A) Schematic setup for sand impact abrasion test and (B) water droplet impact test. SEM images of filters (C) with and (D) without spraying chitosan solution on wire mesh. Photographs of filters (E) with and (F) without spraying chitosan solution on wire mesh after sand impact abrasion test. (G–I) (G) The long-term PM_2.5_ and PM_10_ removal efficiencies by the air filter under continuous hazardous level of PM pollution. Insert is the demonstration of using transparent filter to shut off PM from the outdoor (left bottle) from entering the indoor (right bottle) environment. (H) SEM and (I) TEM images showing the air filter morphology after 100-h PM capture test.

In a practical application, the mechanical robustness and long-term performance of the nanofibrous filters are of great importance. Here, sand abrasion and waterfall tests were applied for assessment of the mechanical durability of the fibrous membrane on wire mesh substrates. As shown in Figures 6C and 6D, the nanofibers were observed to strongly adhere to the wire mesh after spraying chitosan coating. However, if the fibers are simply placed on substrates, the adhesion between the fibrous membrane and wire mesh will be poor. In this test (Figure 6A), the PDMS/PMMA-chitosan air filter was impinged by 50 g sand grains with diameter of approximately 300 μm from a height of 50 cm for 30 s. There was hardly any damage observed on the sprayed filter surface (Figure 6E) after the sand impingement. However, the filter surface was scratched badly by the falling sand grains and obvious damage appeared on the filter surface without spraying chitosan solution (Figure 6F), indicating the key role that the chitosan solution has played in enhancing the adhesion strength between the fibrous membranes and the mesh wire. In addition, a water drop impact test was carried out for assessment of drop impact stability, as seen in Figure 6B. After releasing approximately 5,000 water droplets (ca. 22 μm each) from a height of 50 cm over the filter surface for about 2 h, the surface presented no damage, suggesting robust mechanical durability (Figures S6C and S6D).

In addition, we have carried out a long-term field test. A PDMS/PMMA-chitosan air filter with 54% transmittance was used to evaluate the long-term performance via a self-made device of PM pollution removal (insert of Figure 6G) under a simulated heavy pollution condition with the PM_2.5_ > 1,500 μg m^−3^ and PM_10_ > 2,000 μg m^−3^. After testing for 100 h, the collection bottle still looked clear, suggesting a very low level of the PM_2.5_ particle passing through (typically less than 50 μg m^−3^). As displayed in Figure 6G, such filters could still maintain a high level of removal efficiency for PM_2.5_ over 97% and PM_10_ over 99% after a 100-h test. The SEM and TEM images in Figures 6H and 6I display the surface morphology of PM attached to the nanofibers. The outer surface was covered with a layer of light amorphous organic matters, thus leading to an obvious increase in the diameter of the nanofibers from ∼300 nm to ∼1 μm. This test further confirms the excellent efficacy of the PDMS/PMMA-chitosan air filters.

### Antibacterial Performance

The antibacterial ability of fibrous membranes has been investigated via the colony counting method. In Figures S7A–S7H, pictures of bacterial colony spreading on the agar plate of the blank, PDMS/PMMA fiber membrane, chitosan membrane, and composite membrane have been displayed. Figure S7I indicates the antibacterial efficiency by counting the bacterial colony. Obviously, the amount of *S. aureus* and *E. coli* colonies with the blank and PDMS/PMMA membranes was much larger than that with chitosan and composite membranes. To our surprise, while culturing with chitosan membrane, very few bacterial colonies could be detected on the agar plate, indicating up to 97.7% and 96.4% antibacterial efficiency for *S. aureus* and *E. coli*, respectively. However, the antibacterial efficiency was just 1.2% for *S. aureus* and 3.5% for *E. coli* from PDMS/PMMA membranes, implying almost no antibacterial ability. For the composite membranes, the antibacterial activity was 96.5% for *E. coli* and 95.2% for *S. aureus* owing to the presence of the chitosan component. The antibacterial performance of chitosan-based materials could be ascribed to the surface charge, which leads to the permeation of the cytoplasmic membrane through electrostatic interaction between the bacteria and antibacterial materials, leading to the leakage of intracellular constituents and the death of bacteria. Furthermore, the membrane was capable of inhibiting the reproduction of bacteria by chelating the trace metal (Gu et al., 2018, Wang et al., 2018).

### Conclusions

Versatile and scalable PDMS/PMMA-chitosan air filters have been successfully fabricated by electrospinning. Synergistic effect of small diameter and the polar chemical functional groups from the outer surface of chitosan fibers has made the fibrous membrane an ideal candidate for efficiently capturing PM particles. The superhydrophilic chitosan fibers play the role of enhanced removal capability, whereas the superhydrophobic PDMS/PMMA fibers serve as a blocker to prevent water from accumulating inside the membrane. The obtained fiber filter membrane is capable of holding an outstanding capture efficiency for PM particles (PM_2.5_ > 98.0%, and PM_10_ > 98.4%), a low pressure drop of 21 Pa, and a high flow rate of 1.9 m s^−1^ after 60 min in a high-humidity atmosphere. In addition, the electrospun transparent fibrous membrane with 54% optical transmittance could be continuously applied in an extremely hazardous environment for as long as 100 h with a constant removal efficiency. The filter was also able to maintain high removal efficiency after five cleaning cycles. Besides, the nanofibrous filters also present excellent antibacterial ability because of the chitosan component. Such physical design and bilayer structure pave the way for novel nanofiber materials applicable to a variety of fields, especially for realizing a fresher indoor environment.

### Limitations of Study

We have presented transparent antibacterial PDMS/PMMA-chitosan air filters for effective PM removal, especially in a high humidity atmosphere. However, the fibrous membranes would not be well preserved under extreme weather, such as typhoon, rainstorm, and hailstone. It would be more promising if the filters are robust to resist the harsh weather and retain sustainable PM capturing efficiency.

## Methods

All methods can be found in the accompanying Transparent Methods supplemental file.

## Supplemental Information

Document S1. Transparent Methods and Figures S1–S7
