# Supplementary material for: Transparent Antibacterial Nanofiber Air Filters with Highly Efficient Moisture Resistance for Sustainable Particulate Matter Capture
Source: iScience. 2019 Jul 19;19:214–23. doi: 10.1016/j.isci.2019.07.020 (PMC6698280; doi:10.1016/j.isci.2019.07.020)
Supplement: Document S1. Transparent Methods and Figures S1–S7 [file mmc1.pdf]

ISCI, Volume *19*

Supplemental Information

Transparent Antibacterial Nanofiber Air Filters with Highly Efficient Moisture Resistance for Sustainable Particulate Matter Capture

Hui Liu, Jianying Huang, Jiajun Mao, Zhong Chen, Guoqiang Chen, and Yuekun Lai

Transparent Methods

Materials: Poly(methylmethacrylate) (PMMA, M_W = 3.3 × 10^4 g mol^{-1}) was acquired from Shanghai Titan Science and Technology Corporation. Polydimethylsiloxane (PDMS) prepolymer (Sylgard 184A) and the curing agent (Sylgard 184B) were obtained from Dow Corning Corporation. N,N-dimethylformamide (DMF) was purchased from Shanghai Chemical Reagents Co, Ltd., China. Tetrahydrofuran (THF) was from Suzhou Aladdin Biotechnology Corporation. Chitosan (M_W = 1.9 × 10^4 g mol^{-1}) was purchased from Shanghai Aladdin Bio-Chem Technology Company. Poly(ethylene oxide) (PEO) (M_W = 3 × 10^5 g mol^{-1}) was purchased from Sigma-Aldrich. Commercial detergent was obtained from Guangzhou Libai Enterprise Group Co. Ltd; Wire mesh (1.05 mm × 1.05 mm) was purchased from commercial source; E. coli (ATCC8739) and S. aureus (ATCC 6538), buffered saline solution (PBS) (7.16 g L^{-1} $Na_2HPO_4 \cdot 12H_2O$, 1.36 g L^{-1} KH_2PO_4), nutrient broth and agar powder were obtained from Sinopharm Chemical Reagent Co., Ltd. (China). All chemicals were used without further modification.

Electrospinning of nanofibrous membrane: To obtain PMMA/PDMS solution, 15 wt% PMMA particles and 15 wt% PDMS were dissolved in THF and DMF mixed solution (v:v=1:1) at 50 ℃ under ultrasonication for 24 h. To prepare chitosan solution, 2 g chitosan and 3 g PEO were respectively dissolved in 100 mL 0.5 mol L^{-1} acetic acid solution, subsequently followed by mixing them with weight ratio of 8:1. Then, 1.8 g dimethylsulphoxide (DMSO) and 0.1 g of Triton X-100TM were added. The mixture was then electromagnetically stirred for 24 h to ensure complete dissolution. The solution was centrifuged to remove air bubbles before use. Here, Triton X-100TM and dimethylsulphoxide (DMSO) acted as a nonionic surfactant and a co-solvent to improve processing condition and to increase fiber yields by relaxing chitosan chain entanglement, respectively. The electrospinning solution was firstly sucked into a syringe of 5 ml and then

extruded from 22 G needle tip with a pump rate of 0.2 mL h^{-1} via a syringe pump driven by a voltage supply at 20 kV. The wire mesh with wire diameter of 0.27 mm and mesh size of 0.85 mm assembled on an aluminum foil, the grounded collector. The distance between the needle and the collector was 15 cm. We designed the PDMS/PMMA-chitosan fibrous filters by setting chitosan fibers as the inner layer and PDMS/PMMA fibers as the outer layer. Different ratios of PDMS/PMMA and chitosan fibers can be realized through the control of pump rate and electrospinning time.

PM removal measurement: As shown in Figure S3, PM pollution filtration was carried out in a home-built device. The sample filters were placed in the middle of the box. The generated smoke by the burning of incense and an electric fan were respectively placed on two sides of the device, ensuring the air flow with PMs to travel through the filter. A transparent plastic bag was used to collect the purified air behind fan, and the number and concentration for PM particles with and without the filter was measured using a high-sensitivity particle counter (CEM DT-9880M). The detection was finished before the collection bag has reached its maximum capacity. The PM removal efficiency of the filters was obtained according to the following equation:

$$E_{PM}\ (\%) = (C_b - C_a)/C_b \times 100\% \tag{1}$$

where C_b (μg m^{-3}) and C_a (μg m^{-3}) refer to the PM concentrations before and after the filtration, respectively.

Antibacterial performance: The antibacterial property of fibrous air filters was investigated by colony counting method. S. aureus and E. coli were used as the model bacteria. Briefly, the fresh bacteria solution were obtained by inoculating the model bacteria in 20 mL Lysogeny Broth (LB) liquid medium and culturing them in an incubator shaker which was set at 120 rpm and 37 ℃ for one day. Afterwards, the bacterial suspension was diluted with LB liquid medium for 100 times

and then with PBS for 100 times. Subsequently, after sterilization via ultraviolet radiation, the fibrous filters (0.75 g, 1 cm ×1 cm) were added into a flask with 70 mL the PBS and 5 mL bacteria solution descried above to obtain a mixture, which was then cultured in an incubator shaker at 150 rpm and 25 ℃ for 24 h. After the growth phase, the bacteria suspension was diluted for 10 times and then dispersed in the prepared agar plates in the Petri dishes, which were then placed in the biochemical incubator at 37 ℃ for 24 h. The number of survival bacteria colonies in the agar plate was counted, and the antibacterial efficiency is calculated by equation (1):

$$\mathrm{E}_{Ab}\ (\%) = (\mathrm{C}_0 - \mathrm{C}_1)/\mathrm{C}_0 \times 100\% \qquad (2)$$

where C_0 and C_1 refer to the bacterial concentration after the culturing course with the blank sample and the PDMS/PMMA-chitosan membrane, respectively.

Characterizations: The field emission scanning electron microscopic (FESEM) images were obtained busing Hitachi S-4800 at 3.0 kV to observe the surface morphology of fibrous filters before and after filtration. Transmission electron microscopic (TEM) images were taken under FEI Tecnai G-20. The energy dispersive spectroscopy (EDS) device attached to the FESEM was used to analyze elemental compositions. The contact angles were measured via an optical contact angle measurement system (Krüss DSA100). A surface tension instrument (Dataphysics DCAT11, Germany was used to measure the liquid adhesive force. A Nicolet 5700 Fourier transform infrared (FTIR) spectrometer was used to analyze the chemical bonding information. A Cary 5000 UV-Vis spectrophotometer was applied in the visible range for investigation of transparency for the nanofiber filters. A differential pressure gauge (Testo 510, Germany) and a flowmeter (Testo 450-V1, Germany) were employed to measure the pressure drop and the flow rate, respectively.

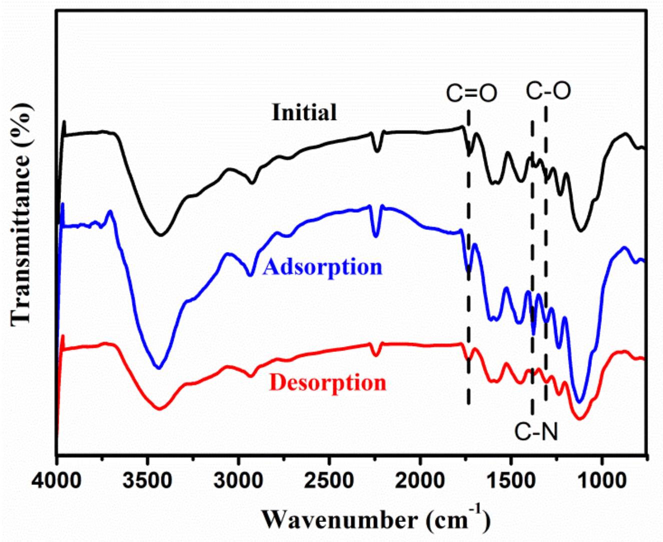

Figure S1. Spectra are presented as FT-IR analysis, Related to Figure 1. FT-IR spectra of PDMS/PMMA-chitosan fibers before PM capture, after PM attached and after washing.

(a)

Sample	Element	Weight %	Weight % σ	Atomic %
Initial	Carbon	53.944	1.450	69.304
	Oxygen	24.037	1.319	23.184
	Silicon	12.284	0.486	6.749
Adsorption	Carbon	53.744	0.818	69.428
	Nitrogen	5.511	1.235	1.614
	Oxygen	21.484	0.489	21.958
	Silicon	12.125	0.207	6.458
Desorption	Carbon	57.449	0.299	72.030
	Oxygen	25.983	0.290	23.244
	Silicon	11.561	0.081	4.363
	Gold	5.007	0.166	0.364

(b)

Sample	Element	Weight %	Weight % σ	Atomic %
Initial	Carbon	54.246	1.023	62.177
	Nitrogen	3.304	1.641	5.311
	Oxygen	37.702	0.781	32.175
Adsorption	Carbon	54.717	0.840	64.060
	Nitrogen	7.264	1.286	6.175
	Oxygen	34.171	0.591	29.495
Desorption	Carbon	52.303	1.134	63.013
	Nitrogen	3.262	1.867	3.370
	Oxygen	36.525	0.878	33.036

Figure S2. Data are represented as EDS analysis, Related to Figure 1. EDS spectrum of (a) PDMS/PMMA fibers and (b) chitosan fibers before PM capture, after PM attached and after washing.

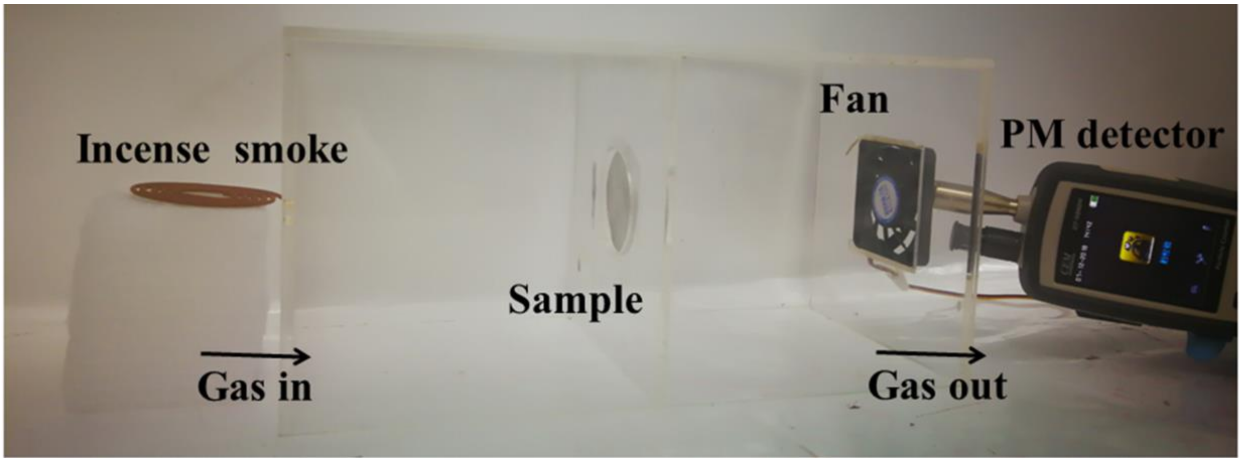

Figure S3. Photograph of PM removal device, Related to Figure 1. Photo images of the PDMS/PMMA-chitosan air filters during PM removal test through home-built device.

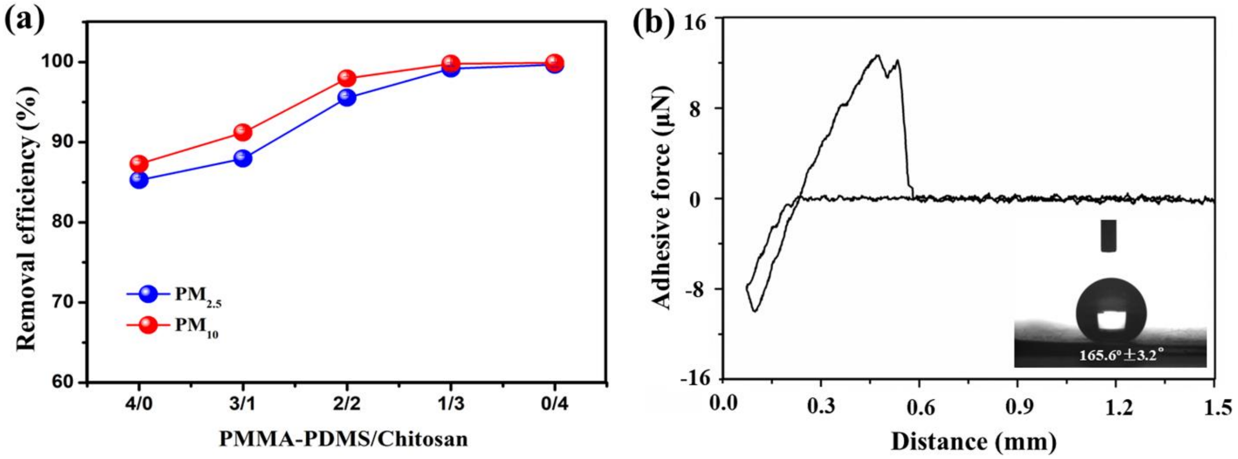

Figure S4. The comparasion of filters with different rations and superhydrophobic performance of PDMS/PMMA nanofibers, Related to Figure 3. (a) Removal efficiency of PDMS/PMMA-chitosan nanofibers with different PDMS/PMMA to chitosan fiber ratios. (b) Adhesive force and contact angle of PDMS/PMMA nanofibers.

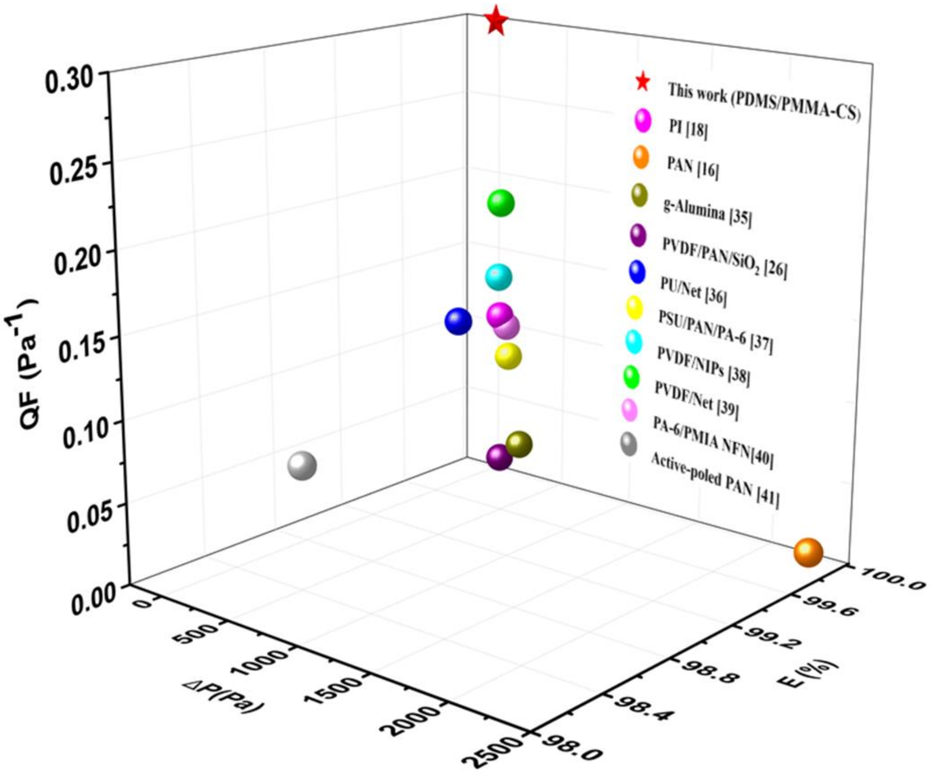

Figure S5. Filtration performance comparison between this work and other reported fibrous filters, Related to Figure 4. It shows that PDMS/PMMA-CS nanofiber filters have the best air filtration performance in terms of quality factor, pressure drop and removal efficiency.

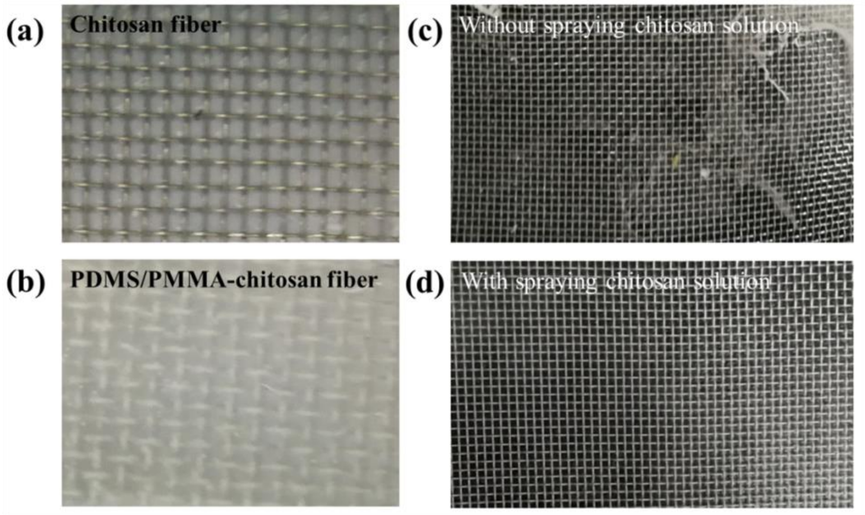

Figure S6. Photographs of nanofibrous membranes after the humidity tests and mechanical tests, Related to Figure 3 and Figure 6. Photo image of air flow pass through (a) pure chitosan nanofibers filters and (b) PDMS/PMMA-chitosan nanofibers filters under humidity conditions. Photo images of filters (c) without and (d) with spraying chitosan solution on wire mesh after water droplet impact test.

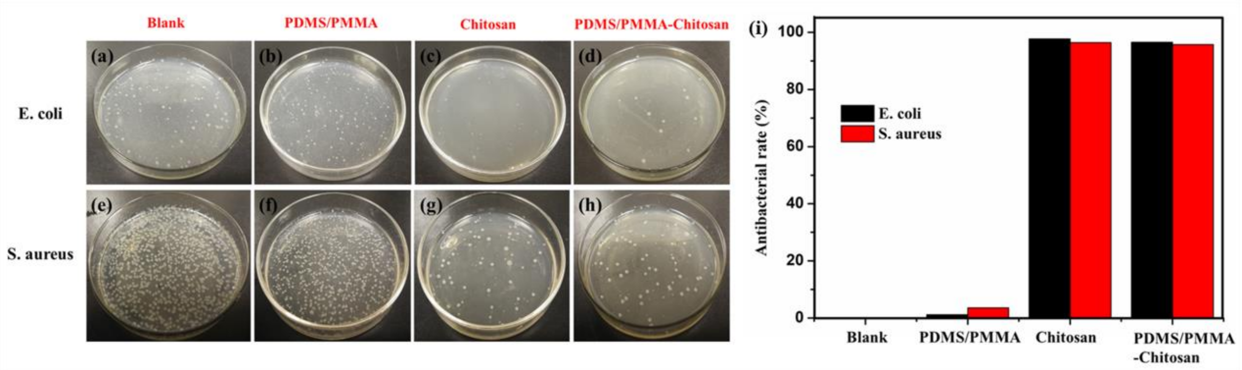

Figure S7. The antibacterial ability of fibrous membranes, Related to Figure 6. Photographs of (a-d) E. coli and (e-h) S. aureus colonies on the agar plate related to the blank, PDMS/PMMS membrane, chitosan membrane and PDMS/PMMA-chitosan blend membrane. (i) antibacterial rate by counting the bacteria colony. It indicates that the nanofibrous filters present excellent antibacterial ability because of the chitosan component.
